# A gene expression microarray for *Nicotiana benthamiana* based on de novo transcriptome sequence assembly

**DOI:** 10.1186/s13007-016-0128-4

**Published:** 2016-05-20

**Authors:** Michal Goralski, Paula Sobieszczanska, Aleksandra Obrepalska-Steplowska, Aleksandra Swiercz, Agnieszka Zmienko, Marek Figlerowicz

**Affiliations:** Institute of Bioorganic Chemistry, Polish Academy of Sciences, Noskowskiego 12/14, 61-704 Poznan, Poland; Institute of Plant Protection – National Research Institute, Wladyslawa Wegorka 20, 60-318 Poznan, Poland; Institute of Computing Science, Poznan University of Technology, Piotrowo 2, 60-965 Poznan, Poland

**Keywords:** *Nicotiana benthamiana*, Microarray, Coding strand, Leaf and root transcriptome

## Abstract

**Background:**

*Nicotiana benthamiana* has been widely used in laboratories around the world for studying plant-pathogen interactions and posttranscriptional gene expression silencing. Yet the exploration of its transcriptome has lagged behind due to the lack of both adequate sequence information and genome-wide analysis tools, such as DNA microarrays. Despite the increasing use of high-throughput sequencing technologies, the DNA microarrays still remain a popular gene expression tool, because they are cheaper and less demanding regarding bioinformatics skills and computational effort.

**Results:**

We designed a gene expression microarray with 103,747 60-mer probes, based on two recently published versions of *N. benthamiana* transcriptome (v.3 and v.5). Both versions were reconstructed from RNA-Seq data of non-strand-specific pooled-tissue libraries, so we defined the sense strand of the contigs prior to designing the probe. To accomplish this, we combined a homology search against *Arabidopsis thaliana* proteins and hybridization to a test 244k microarray containing pairs of probes, which represented individual contigs. We identified the sense strand in 106,684 transcriptome contigs and used this information to design an Nb-105k microarray on an Agilent eArray platform. Following hybridization of RNA samples from *N. benthamiana* roots and leaves we demonstrated that the new microarray had high specificity and sensitivity for detection of differentially expressed transcripts. We also showed that the data generated with the Nb-105k microarray may be used to identify incorrectly assembled contigs in the v.5 transcriptome, by detecting inconsistency in the gene expression profiles, which is indicated using multiple microarray probes that match the same v.5 primary transcripts.

**Conclusions:**

We provided a complete design of an oligonucleotide microarray that may be applied to the research of *N. benthamiana* transcriptome. This, in turn, will allow the *N. benthamiana* research community to take full advantage of microarray capabilities for studying gene expression in this plant. Additionally, by defining the sense orientation of over 106,000 contigs, we substantially improved the functional information on the *N. benthamiana* transcriptome. The simple hybridization-based approach for detecting the sense orientation of computationally assembled sequences can be used for updating the transcriptomes of other non-model organisms, including cases where no significant homology to known proteins exists.

**Electronic supplementary material:**

The online version of this article (doi:10.1186/s13007-016-0128-4) contains supplementary material, which is available to authorized users.

## Background

The multiple genome sequencing projects undertaken during the last decade constituted the basis for integrated, whole-genome studies of genes, gene functions and regulatory mechanisms in various organisms. Analysis of the cell’s transcriptome composition and dynamics in response to specific stimuli provides important insights into the complexity of the gene regulatory network and key genetic players [1–3]. Currently, the most commonly used techniques for genome-wide expression studies are DNA microarrays and high throughput RNA sequencing (RNA-Seq) [4]. The latter technique directly reveals the sequence of transcripts and is becoming increasingly popular, as a result of continuous improvements in both the sequencing technology and the data analysis software. This increase has been marked by the development of sequencing centers and large consortia focused on specific organisms (Rice Genome Annotation Project, 1001 Arabidopsis Genomes Project, The Maize Genome Sequencing Consortium, to name just a few). These communities work on developing and standardization of protocols to facilitate aggregating and comparison of various datasets. Current RNA-Seq applications involve assembly of the transcriptome, with or without the reference genome information, gene discovery and expression analysis, identification of unknown exon junctions and alternative transcripts, measuring allele-specific expression and many more [5–8]. On the contrary, microarrays can only derive information on targets that are actually represented by the microarray probes and are sensitive to cross-hybridization, as well as display poor signal resolution and increased variation at low signal intensities [9, 10]. Despite these drawbacks, the results generated on microarray platforms are concordant with those obtained with RNA-Seq [11, 12]. Additionally, thousands of studies performed over the past decades proved that the microarrays reflect the transcriptome composition with high fidelity and that they are a rich source of biologically valuable information. Since their introduction, microarrays have been effectively used in searching for disease markers [13], alternative splicing [14], gene function prediction [15], identification of transcriptionally active regions of the nuclear, mitochondrial and chloroplast genomes [16–18] and many other applications. The microarray experiments are still much cheaper than RNA-Seq, not only regarding the price of consumables and reagents but also the computational and human resources required for data analysis and storage. The latter are often underestimated when calculating the real costs of high-throughput sequencing experiments [19]. Remarkably, extracting biological information from the RNA-Seq data requires combining computational skills with deep knowledge of the problem of interest, typically by the close cooperation of experts in each of those fields. Therefore, sequencing-based experiments may pose a substantial challenge for individual laboratories. With the small size of the resulting datasets and the relatively easy data analysis, DNA microarrays are still an attractive alternative to RNA-Seq for a variety of studies, e.g., focused on differential analysis of known genes in the conditions of study and in time-course studies, where a large number of samples are to be processed and compared in a repeatable manner. We surveyed the gene expression profiling experiments for *Arabidopsis* and rice, deposited in Gene Expression Omnibus database in years 2012–2015. Those which utilize DNA microarrays constantly outnumber the sequencing-based studies (Additional file 1: Figure S1). Even taking into account the delays in publishing results of research projects, this comparison proves that the DNA microarrays are still used for measuring gene expression changes.

*Nicotiana benthamiana* is a plant model widely used in many laboratories around the world, especially in plant-pathogen interaction studies, due to the ease of infection by a large number of plant viruses [20–23], viroids [24], bacteria [25, 26] and fungi [27, 28]. However, the numbers of *N. benthamiana* gene expression profiling results available in public databases, such as Gene Expression Omnibus (GEO) or ArrayExpress, are surprisingly low. One possible reason for the low abundance is the lack of a microarray platform dedicated to *N. benthamiana*. The detailed design of the only *N. benthamiana*-specific oligonucleotide microarray described so far [29] based on Expressed Sequence Tags data has not been revealed (this information is available on demand from the authors [29]) and the microarray has not been widely used. Moreover, it was produced in the NimbleGen technology, which was discontinued and the NimbleGen microarrays are no longer available to purchase. Nearly 98 % of microarray experiments for *N. benthamiana* reported in GEO were carried out with microarrays specific to another *Solanaceae* species, thus employing a so-called cross-species hybridization (CSH) approach. This approach gained considerable popularity before RNA-Seq methods were introduced on a broad scale and was successfully utilized to profile gene expression in multiple organisms with limited sequence information as well as to extract valid biological information [30–33]. However, it was admitted that CSH analyses suffer from several limitations, which significantly reduce the amount of information that can be derived from the microarrays. These limitations include: higher proportion of genes with no detectable signal (due to lack of the target matching the probe), higher risk of cross-hybridization of transcripts that have similar (but not perfect) homology to the microarray probe, and the lack of probe representation for genes specific to the organism of interest. Also, several authors reported that CSH is characterized by lower mean signal intensity and disturbed spot morphology in comparison with single species hybridization, as a result of weaker binding of targets to their non-perfectly matching probes [34, 35]. All these disturbances affect the overall quality of microarray data and complicate the analysis steps. Published reports on sequencing the *N. benthamiana* genome [36, 37] and, more recently, on assembling the *N. benthamiana* transcriptome from RNA-Seq data [38, 39] now enable researchers to perform *N. benthamiana*-oriented studies in the broader, genomics context.

To facilitate these types of studies, we designed an oligonucleotide microarray (Nb-105k microarray) based on the transcriptomes assembled de novo from the short reads by Australian & New Zealand Consortium and described in detail previously [38, 39]. As those transcriptomes were derived from the non-strand-specific libraries, we used bioinformatics and experimental approaches to ensure the correct orientation of the probes on the final microarray. Our microarray design has been made public via the Agilent eArray platform. In our experience, the Nb-105k microarray showed excellent performance and high sensitivity when employed for gene expression profiling in leaves *versus* roots. We predict that it will become an appreciated and useful gene expression analysis resource for the *N. benthamiana* research community.

## Results

### Determination of the sense cDNA strand in *N. benthamiana* transcriptome v.3 unigenes

To facilitate the gene expression studies in *N. benthamiana*, we decided to design a custom microarray tool which will represent the entire *N. benthamiana* transcriptome and will be compatible with a widely employed Agilent technology platform. The microarray was primarily based on transcriptome v.3, which represented the most contemporary and comprehensive source of *N. benthamiana* transcripts available at the time our work was initiated. The transcriptome v.3 was previously created with the effort of the Australian & New Zealand Consortium, by assembling 193 million short reads generated from the RNA-Seq analysis of nine different *N. benthamiana* tissues on an Illumina sequencing platform using Abyss v1.3 and Trans-Abyss v1.1 [38]. It consists of a representative set of 119,014 unigenes with an average size of 795 bp (the longest contig size was 14,845 bp) and an extended dataset of 237,340 unique transcripts, including all spliced isoforms detected for each gene.

**Fig. 1:**
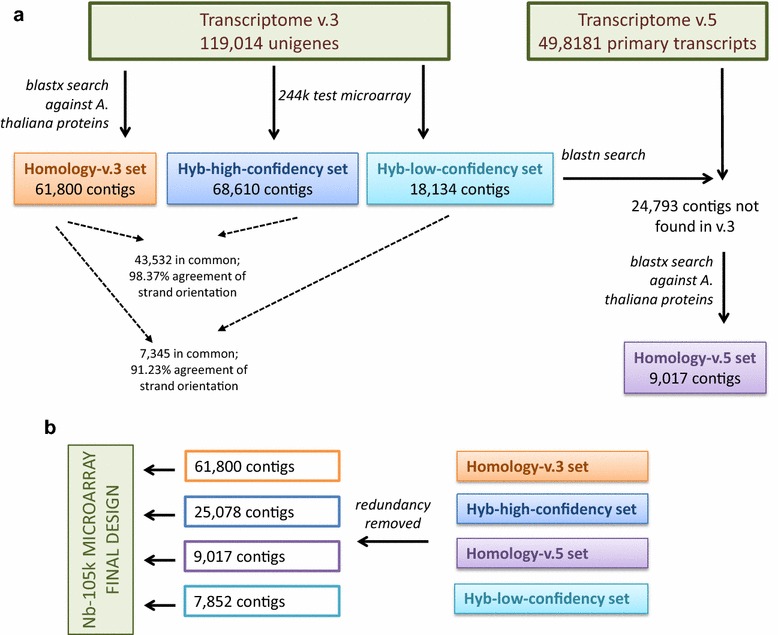
Schematic representation of the Nb-105k microarray design process. **a** Coding strand prediction and sequence selection; **b** removing redundancy and combining the datasets. See main text for the details

We expected random orientation of the unigenes because they were assembled de novo from reads that originated from non-strand-specific libraries. This posed a problem, as the oligonucleotide probe design step requires a priori knowledge of the transcript orientation. The probes targeting non-coding strands will be useless, as they will not recognize the intended transcript (represented by fluorescently labeled single-stranded cDNA/cRNA). To determine the coding strand, we performed homology searches on the amino acid level against *Arabidopsis thaliana* reference protein set. Matches were found for 61,800 *N. benthamiana* unigenes (Homology-v.3 set, see Fig. 1) and their orientation was chosen accordingly. Of the unigenes, 50.4 % needed conversion to their reverse complement, confirming our expectations regarding strand randomness introduced by the sequencing and de novo assembly process.

**Fig. 2:**
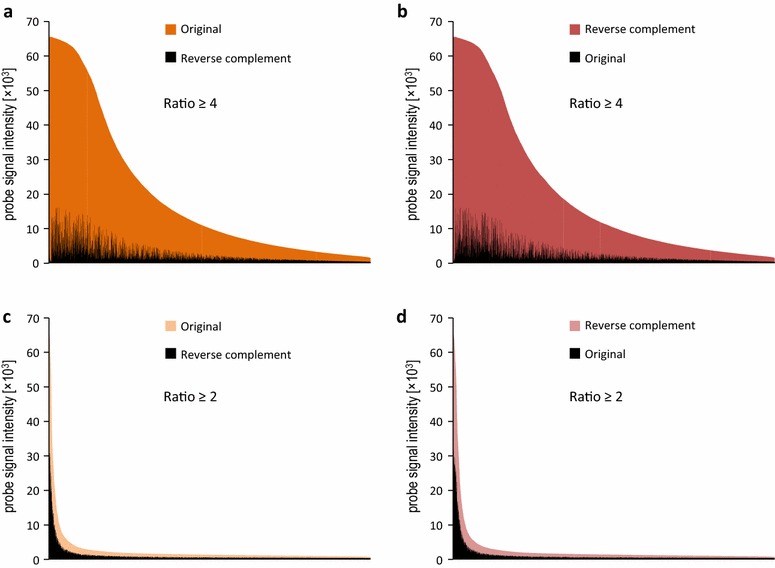
Detection of the sense strand in v.3 unigenes by hybridization to the 244k microarray. The signal intensities of probes representing each contig in both orientations were compared, and the probe with the stronger signal was defined as the sense one. **a**, **b** Probes selected for the Hyb-high-confidency set, with an intensity ratio ≥4 and an identical (**a**) or reverse complement (**b**) orientation relative to the v.3 unigene sequence; **c**, **d** probes selected for the Hyb-low-confidency set, with an intensity ratio ≥2, but less than 4 and an identical (**c**) or reverse complement (**d**) orientation relative to the v.3 unigene sequence. Probes identified as sense are marked in *orange*/*red*; the antisense pairs are in *black*

Homology searches provided the strand orientation for approximately half of the unigenes. To increase this number, we turned to experimental detection of the coding strand. To accomplish this, we designed a test 244k microarray with pairs of probes representing 118,934 unigenes in both orientations (80 shortest unigenes from the v.3 transcriptome were not included, due to the microarray capacity limitations). The microarray was hybridized to a labeled RNA pool from various tissues and experimental treatments of *N. benthamiana*. The sense probe was then identified for each unigene as being the only one from the pair which produced a detectable microarray signal or had a stronger signal. We arbitrarily set the relative signal intensity of the expressed strand to be at least 4 times higher than its reverse complement to ensure high confident predictions (Fig. 2a, b). The acceptance criteria were fulfilled by 68,610 microarray probes (Hyb-high-confidency set, see Fig. 1), of which 49.1 % were in the same orientation as the transcriptome v.3 unigenes, in accordance with the expected strand randomness of the unigenes. Next, we analyzed unigenes for which the probe signal intensity ratio was not less than two but below four (Fig. 2c, d). We identified 18,134 such cases (Hyb-low-confidency set, see Fig. 1), of which 50.8 % were in the same orientation as the transcriptome v.3 contigs.

We also compared the hybridization-based and homology-based predictions. *A. thaliana* protein matches were available for 43,532 unigenes from the Hyb-high-confidency set and for 7345 unigenes from the Hyb-low-confidency set. Homology predictions identified the same coding strand as the hybridization-based approach, for 98.37 and 91.23 % of them, respectively. We concluded that the hybridization-based approach allowed us to predict the coding strand with high accuracy and that both Hyb-high-confidency and Hyb-low-confidency sets were composed of reliable data. In summary, we defined the coding strand for an additional 32,930 unigenes, with no homology-based predictions. Altogether, both approaches (homology-based and hybridization-based) defined the correct orientation of 79.6 % of the v.3 contigs. Whenever the two methods resulted in contradictory predictions, we chose the coding strand from the homology data.

### Determination of the sense cDNA strand in *N. benthamiana* transcriptome v.5 contigs

After initiating our microarray method development, a newer version of the *N. benthamiana* transcriptome (v.5) was published [39]. The updated version was generated by increasing the amount of sequenced data and combining four de novo assemblers (TransAbyss, Trinity, SOAPdenovo-Trans, and Oases). This approach generated much longer sequence assemblies (with a mean length 1674 bp). The new transcriptome consists of 49,818 primary transcripts (representative models, usually with the longest sequence) and 184,708 alternative transcripts (cDNA isoforms other than in the primary set). We compared v.5 primary transcripts with sequences from the Homology-v.3 set, Hyb-high-confidency set and Hyb-low-confidency set and selected those without any significant matches. We then used their translated sequences in a homology search against *A. thaliana* proteins, as described above. As a result, we supplemented our dataset with 9017 additional contigs, derived from transcriptome v.5, for which the coding strand could be inferred by homology (Homology-v.5 set, see Fig. 1).

### Nb-105k microarray design and gene expression analysis

The four sets of sequences with defined orientations (Homology-v.3, Homology-v.5, Hyb-high-confidency and Hyb-low-confidency) were used to create the final microarray (Fig. 1). The Nb-105k microarray includes 103,747 oligonucleotide probes representing *N. benthamiana* contigs and 1325 standard Agilent positive and negative controls. The performance of the new microarray was verified by comparing the gene expression in leaves vs roots of *N. benthamiana* plants. High quality data were obtained using four biological replicates (see “Methods” section). By applying restrictive filters (including a mean intensity, A_mean_, of at least 6, an FDR-corrected p value < 0.0005, and a relative difference in expression of at least fourfold), we obtained 6643 probes that indicated differential gene expression with a high level of confidence. Of these, 4478 showed up-regulation and 2165 showed down-regulation in leaves.

**Fig. 3:**
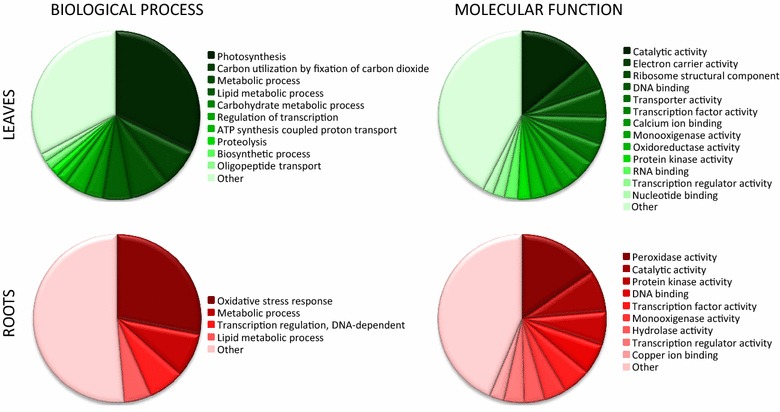
Gene ontology representation of genes differentially expressed in leaves and roots of *N. benthamiana* identified with the Nb-105k microarray. GO terms represented by less than ten genes were combined into the “Other” category

We used available Gene Ontology (GO) information for rapid verification of the biological contents within the differentially expressed gene sets (Fig. 3). GO annotations were available for 22 % of the genes that were up-regulated in leaves. The main categories in this set encompassed genes involved in photosynthesis-related processes and functions. Among the genes that showed up-regulation in roots, 19 % possessed GO annotations. The main biological process represented in this dataset was the stress-response, and the main represented molecular function was peroxidase activity. Based on these analyses, we concluded that the Nb-105k microarray was able to correctly reflect the transcriptome specificities in the two organs under study.

### Verification of v.5 contig assembly using gene expression data generated with Nb-105k microarray

The Nb-105k microarray was designed to contain one probe per contig. However, a comparison of the two transcriptome versions found that multiple v.3 unigenes (from 2 to 173) matched the same v.5 primary transcript. One example was the v.5 primary transcript Nbv5tr6241943, matched by 63 v.3 unigenes, each represented by an individual microarray probe. Nbv5tr6241943 is annotated as Ribulose bisphosphate carboxylase (RuBisCO) small chain 8B. The respective v.3 unigenes covered the whole range of its length (Additional file 1: Figure S2A) and possessed various functional annotations (mostly annotated as RuBisCO chains: 1, 2A, 2B, 3, 3B, 8B). In our microarray experiment, all were up-regulated in leaves (20–317 times, Additional file 1: Figure S2B) and all but one fulfilled the restrictive statistical significance criteria. However, another cluster of 17 v.3 unigenes displayed variability in expression levels even though all of them matched the same v.5 primary transcript Nbv5tr6230285. Close inspection of Nbv5tr6230285 sequence, which is annotated as Uncharacterized RNA methyltransferase pc1998, revealed that it is probably a chimeric contig. Its 5′ end matches a conserved protein domain found in the AdoMet-dependent methyltransferases superfamily (which is encoded in open reading frame +3) and its 3′ end matches a domain of protease HtpX (which is encoded in open reading frame −3) (Additional file 1: Figure S3A). The mapping and relative orientations of v.3. unigenes (that had their coding strands defined by homology searches and hybridization to the 244k test microarray) clearly revealed that only the 3′ part of the Nbv5tr6230285 contig belongs to the gene that is up-regulated in leaves (Additional file 1: Figure S3B). When used in this manner, the gene expression data obtained with the Nb-105k microarray may also aid the improvement of *N. benthamiana* transcriptome annotation.

## Discussion

Despite the increasing availability of genomic and transcriptomic data for many species, the need for bioinformatics skills and access to large computational resources often result in the pool of “big data” being unused by biologists. Gene expression microarrays are relatively easy to explore, even by non-expert users. Dedicated databases (e.g., Genevestigator, Plant Expression Database) provide access to integrated microarray data from multiple sources and experiments, allowing intra- and interspecies comparisons. Here, we described a *N. benthamiana* gene expression microarray (Nb-105k microarray) which reflects the current, advanced state of knowledge regarding the *N. benthamiana* genome and transcriptome. Based on our experience, commercial microarrays for plants that either have not been sequenced or do not have fully annotated genomes (e.g., Agilent Tobacco 4 × 44k or Barley 4 × 44k gene expression microarrays, which are based on 2004–2008 sequence releases) typically include multiple probes that have the incorrect orientation and, as a consequence, do not produce useful data [40, Goralski et al., unpublished]. In the present work, we used a combination of bioinformatics and experimental approaches to detect the sense orientation of the contigs prior to designing the probes. We defined the coding strand for 66.4 % of v.3 transcriptome unigenes using homology searches and for 81.3 % of v.3 transcriptome unigenes using RNA hybridization to the test microarray. The latter approach provided substantially more data and did not require sequence homology to known proteins. The efficiency of this simple, hybridization-based verification was demonstrated by the high level of agreement of outputs generated by the two methods. Ultimately, we defined the sense strand orientation of 97,667 contigs from v.3 transcriptome (82.1 %). Apart from the direct application for the Nb-105k microarray design purposes, the data we obtained will serve as a useful guide for future users of this transcriptome dataset.

Our carefully designed microarray consists of nearly 104,000 probes targeting *N. benthamiana* transcripts from de novo assemblies of RNA-Seq data. As the RNA-Seq libraries were prepared from samples representing mixed tissues, the Nb-105k microarray based on these data can be considered a versatile gene expression tool. Indeed, we observed its high sensitivity in detecting and distinguishing the regulation of genes expressed in roots and leaves of *N. benthamiana* plants. The microarray is mainly based on transcriptome v.3 [33], but we also included additional unique sequences from the more recent transcriptome v.5 [34]. The latter version consists of smaller number of contigs that are much larger, as they were assembled from the combination of multiple assemblies and include more short-read data. For this reason, some of the microarray probes derived from v.3 unigenes may map to the same primary transcript from v.5. As shown in the present study, a comparison of the expression profiles generated with such probes allows for additional experimental verification of the transcriptome assemblies. To this end, the Nb-105k microarray usage can be extended from simple gene expression analysis to increasing the accuracy and completeness of the current and future *N. benthamiana* transcriptomes. On the other hand, the future improvement of *N. benthamiana* genome and transcriptome will likely enable re-designing of the Nb-105k microarray, for example to include probes discriminating between paralogous sequences. (The current version of our microarray was not tested for discriminating between highly similar sequences).

In conclusion, we provided details on the design of an oligonucleotide microarray that reflects the current advanced state of *N. benthamiana* transcriptome research. This, in turn, will allow the *N. benthamiana* research community to take full advantage of microarray techniques for studies of gene expression in this plant.

## Methods

### Plant material

*Nicotiana benthamiana* plants were planted on Jiffy pellets and transferred to pots with soil after 2 weeks. The plants were grown in growth chambers at 22 °C/20 °C (day/night). Two sets of samples were prepared for two stages of our experiment, as follows. For detecting the coding strand using hybridization-based approach we collected roots, leaves, and stems from: 4-week old healthy plants, 10-week old healthy plants, 6-week old plants infected with peanut stunt virus, 6-week old plants infected with tomato torrado virus, 6-week old plants wounded mechanically, 6-week old plants wounded by whitefly *Trialeurodes vaporariorum*. For the analysis of gene expression we collected leaves and roots from four independent 6-week old plants (leaves/roots from one plant constituted one biological replicate). All the material was frozen in liquid nitrogen.

### RNA extraction and labeling

Total RNA was extracted using RNeasy Plant Mini Kit QIAGEN) and DNase-digested with TURBO DNA-free kit (Ambion), according to the manufacturers’ standard protocols. RNA quality was determined using Nanodrop 2000 spectrophotometer and capillary electrophoresis in 2100 Bioanalyzer (Agilent). All samples were of high quality, with A^260^/A^280^ ≥ 2 and no visible signs of degradation when analyzed on a capillary electrophoresis gel. For hybridization-based coding strand prediction the frozen plant material samples were mixed in equal amounts before RNA extraction (“pooled sample”). For gene expression analysis total RNA was extracted from one plant at a time, (leaves and roots separately), constituting a biological replicate: “L1”, “L2”, “L3”, “L4” and “R1”, “R2”, “R3”, “R4”. Labeled cRNA samples were prepared from 200 ng RNA, using a Quick Amp Labeling Kit (Agilent) and quality-checked on a Nanodrop 2000 spectrophotometer.

### Homology searches of transcriptome v.3 unigenes

*Nicotiana benthamiana* unigenes (transcriptome v.3) were downloaded from a server at the School of Molecular Bioscience [40]. Each sequence was used as a query in a BLASTX homology search against the *A. thaliana* reference protein set, with e-value threshold set at 0.001. The best *A. thaliana* hit was then used to check and, if needed, correct the orientation of the query sequences. Whenever the orientation of *A. thaliana* protein matching strand was opposite to the *N. benthamiana* sequence (Plus/Minus or Minus/Plus), the latter was converted into its reverse-complement counterpart.

### Design of the 244k test microarray

Oligonucleotide probes (60-mers) representing v.3 unigenes were designed using the Agilent eArray platform. For each unigene, two probes were designed, one for each sequence orientation. During the design process, the target sequences (all v.3 unigenes, in both orientations) were combined into one reference transcriptome dataset, to ensure maximum probe specificity. Due to limited microarray capacity, pairs of microarray probes were designed for 99.93 % unigenes of the transcriptome v.3 (the longest ones). The resulting 244k microarray was purchased from Agilent.

### Hybridization and analysis of the 244k test microarray

Cy3- and Cy5-labeled cRNA obtained from the same “pooled sample” was hybridized to one 244k test microarray in A1 × 244K hybridization chamber (Tecan) on a HS 4800 Pro automatic station (Tecan), according to the manufacturer’s guidelines regarding Agilent microarrays treatment. A Gene Expression Hybridization Kit (Agilent) and Gene Expression Wash Buffer Kit solutions (Agilent) were used for the hybridization and washing steps, respectively. The intensity data were collected with 4200AL GenePix scanner and processed with GenePix Pro 6.1 software using morphological opening background method. Following a quality check with background intensity plotting functions and background subtraction step (“subtract,” offset = 10), implemented in R/Bioconductor limma package [41] the intensity data collected for the Cy3 and Cy5 channels were plotted against each other. They demonstrated a high linear correlation (R^2^ = 0.955). Therefore, only the data for the Cy5 channel were used in the subsequent comparison of the signal intensities of paired probes (see the Results section). The intensity date were further compared in Microsoft Excel 2010.

### Homology searches of transcriptome v.5 primary transcripts

*Nicotiana benthamiana* primary transcripts (transcriptome v.5) were downloaded from a server at the School of Molecular Bioscience [40]. To compare v.3 and v.5 transcriptomes and to supplement our dataset with the contigs missing from the v.3 assembly, we used each v.3 contig of a previously defined strand orientation to query the v.5 dataset with BLASTN. Matches with an e-value <0.001, a gap opening penalty of 6 and a gap extension penalty of 2 were considered significant. With such parameters, the identity level of successfully aligned sequences exceeded 90 % in 93.9 % of the cases tested. The remaining v.5 sequences (those without matches in v.3 dataset) were used as queries in a BLASTX homology search against the *A. thaliana* reference protein set, with an e-value threshold set at 1,E-03 and strand orientation identified as described in the “Homology searches of transcriptome v.3 unigenes” section.

### Design of the Nb-105k microarrays

The sense probes (60-mers) were designed for each transcript from Homology-v.3, Homology-v.5, Hyb-high-confidency and Hyb-low-confidency sets on an Agilent eArray platform. The same four datasets were also combined into a reference transcriptome. This reference transcriptome was used by the Agilent software for design purposes, to ensure maximum specificity of the probes towards their targets. The microarrays in 2 × 105k format (two microarrays per slide) were then purchased from Agilent.

### Hybridization and analysis of the Nb-105k microarrays

Cy3- and Cy5-labeled cRNA from leaves and roots of *N. benthamiana* plants were hybridized to Nb-105k microarrays in the following dye-swap design: Cy3-“L1” versus Cy5-“R2”, Cy3-“L2” versus Cy5-“R3”, Cy3-“R4” versus Cy5-“L3” and Cy3-“R1” versus Cy5-“L4”. The hybridization was performed in A2 × 105K hybridization chambers (Tecan) on a HS 4800 Pro (Tecan) automatic station, according to the manufacturer’s guidelines regarding Agilent microarrays treatment. A Gene Expression Hybridization Kit (Agilent) and Gene Expression Wash Buffer Kit solutions (Agilent) were used for the hybridization and washing steps, respectively. The intensity data were collected with 4200AL GenePix scanner and processed with GenePix Pro 6.1 software using morphological opening background method. The data were then analyzed with standard analysis pipeline implemented in R/Bioconductor limma package [41], as described previously [42, 43]. Briefly, the background was subtracted from the probe intensity data. The normalization steps involved “loess” within array normalization and “Aquantile” between array normalization. Quality plots (‘MA-plot” and “density plot”) were generated to evaluate the data normalization performance and assess the microarray data accuracy and dynamic range. The analysis of Agilent Spike-in controls was also performed and confirmed that the generated data well reflected the theoretical Cy5/Cy3 RNA ratios across a broad range of template copy numbers. The pre-processed data were used for differential expression analysis by applying a Bayesian linear model. The significance threshold was set at a *p* value <0.0005 (after applying Benjamini and Hochberg’s method to control the false discovery rate), a mean intensity (A_mean_) >6 and at least a fourfold relative expression difference between roots and leaves. Gene Ontology annotations of v.3 transcripts were the same as used in [38]. Raw and normalized gene expression data from this experiment were deposited into the NCBI GEO repository [44] and are accessible via a GEO Series accession number GSE76631.

### Microarray probe mappings and availability

The Nb-105k microarray design is registered in NCBI GEO repository under Platform accession number GPL21307 and is also publically available for ordering from Agilent eArray platform under ID 066813. In this design, each microarray probe is annotated with the original ID of the appropriate contigs from transcriptome v.3 or transcriptome v.5. Whenever the reverse complement of the original sequence was used for probe design (according to the results of sense strand identification performed in the current study), the contig ID has an RC_prefix. To download the original Agilent design files, with probe sequences or to order the Nb-105k microarray, go to https://earray.chem.agilent.com/earray/ then click the “Published Designs” link button on the right and select *N. benthamiana* from the species list.

The final microarray probes were also mapped to transcriptomes v.3 and v.5 to provide the links between two transcriptomes datasets. For this, each probe sequence was used as a query in a megablast similarity search against a local database created for a transcriptome v.3 or v.5, transcriptome, respectively, with the following parameters: e-value <0.0001, word size = 7, 65 % identity, no masking for low complexity sequences. Best hit for each search was reported in Additional file 2: Table S1 in addition to the primary targets (those for which the probes were designed).

## Additional files

10.1186/s13007-016-0128-4 Proportions of array-based (*blue*) and high throughput sequencing-based (*orange*) RNA profiling experiments deposited in GEO Database in 2012-2015 for *Arabidopsis* and rice. **Figure S2.** Gene expression data for v.3 unigenes mapping v.5 primary transcript Nbv5tr6241943. **Figure S3.** Gene expression data for v.3 unigenes mapping v.5 primary transcript Nbv5tr6230285.
10.1186/s13007-016-0128-4 Targets of Nb-105k microarray probes in Transcriptomes v.3 and v.5.

## Abbreviations

RNA-Seq: RNA sequencing
GEO: gene expression omnibus
GO: gene ontology
RuBisCO: ribulose bisphosphate carboxylase
