# Supplementary material for: A gene expression microarray for Nicotiana benthamiana based on de novo transcriptome sequence assembly
Source: Plant Methods. 2016 May 20;12:28. doi: 10.1186/s13007-016-0128-4 (PMC4875705; doi:10.1186/s13007-016-0128-4)
Supplement: Supplementary file 1 — 10.1186/s13007-016-0128-4 Proportions of array-based (blue) and high throughput sequencing-based (orange) RNA profiling experiments deposited in GEO Database in 2012-2015 for Arabidopsis and rice. Figure S2. Gene expression data for v.3 unigenes mapping v.5 primary transcript Nbv5tr6241943. Figure S3. Gene expression data for v.3 unigenes mapping v.5 primary transcript Nbv5tr6230285. [file 13007_2016_128_MOESM1_ESM.pdf]

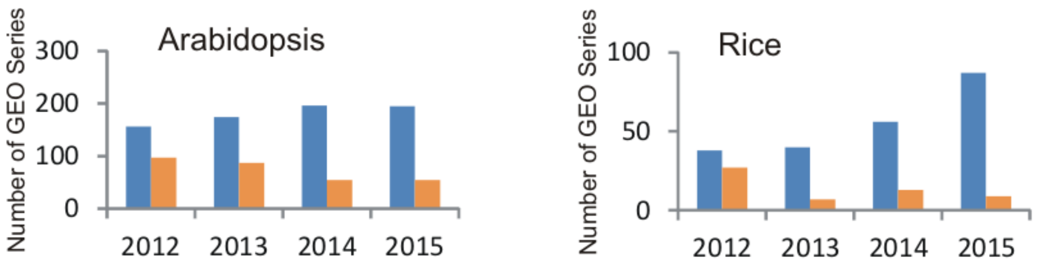

Supplementary Figure S1. Proportions of array-based (blue) and high throughput sequencing-based (orange) RNA profiling experiments deposited in GEO Database in 2012-2015 for Arabidopsis and rice. Gene expression profiling and non-coding RNA profiling experiments deposited as GEO Series were counted (a GEO Series record is an original submitter-supplied record that summarizes an experiment and links together a group of related sample data).

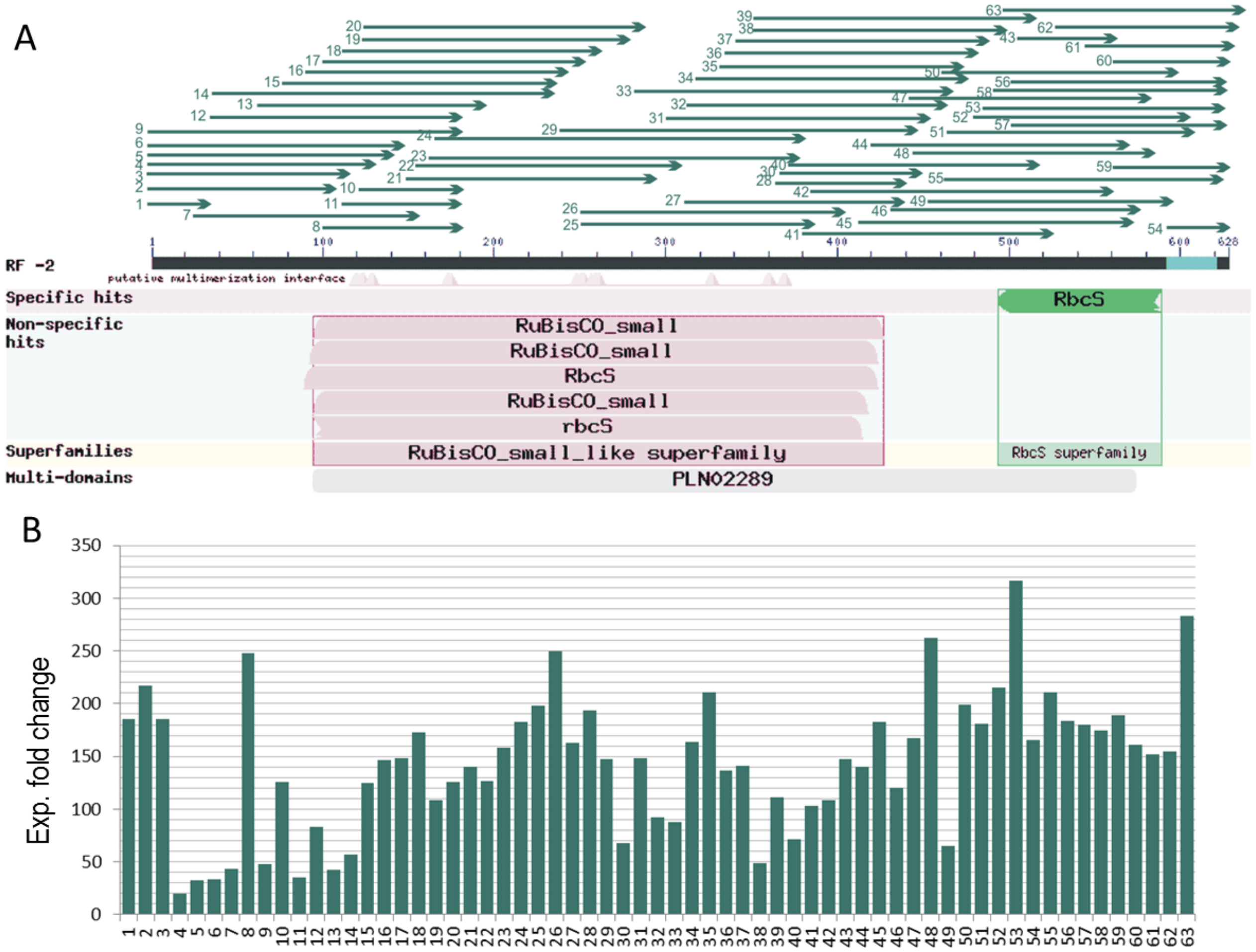

Supplementary Figure S2. Gene expression data for v.3 unigenes mapping v.5 primary transcript Nbv5tr6241943. A, Nbv5tr6241943 encodes conserved RuBisCo and RbcS family protein domains in open reading frame -2. 63 v.3 unigenes of already defined orientation match Nbv5tr6241943 sequence across the whole range of its length; B, Each of 63 v.3 unigenes is represented by a specific probe on Nb-105k microarray. Gene expression data consistently indicate 20-317-fold up-regulation in leaves, compared to roots.

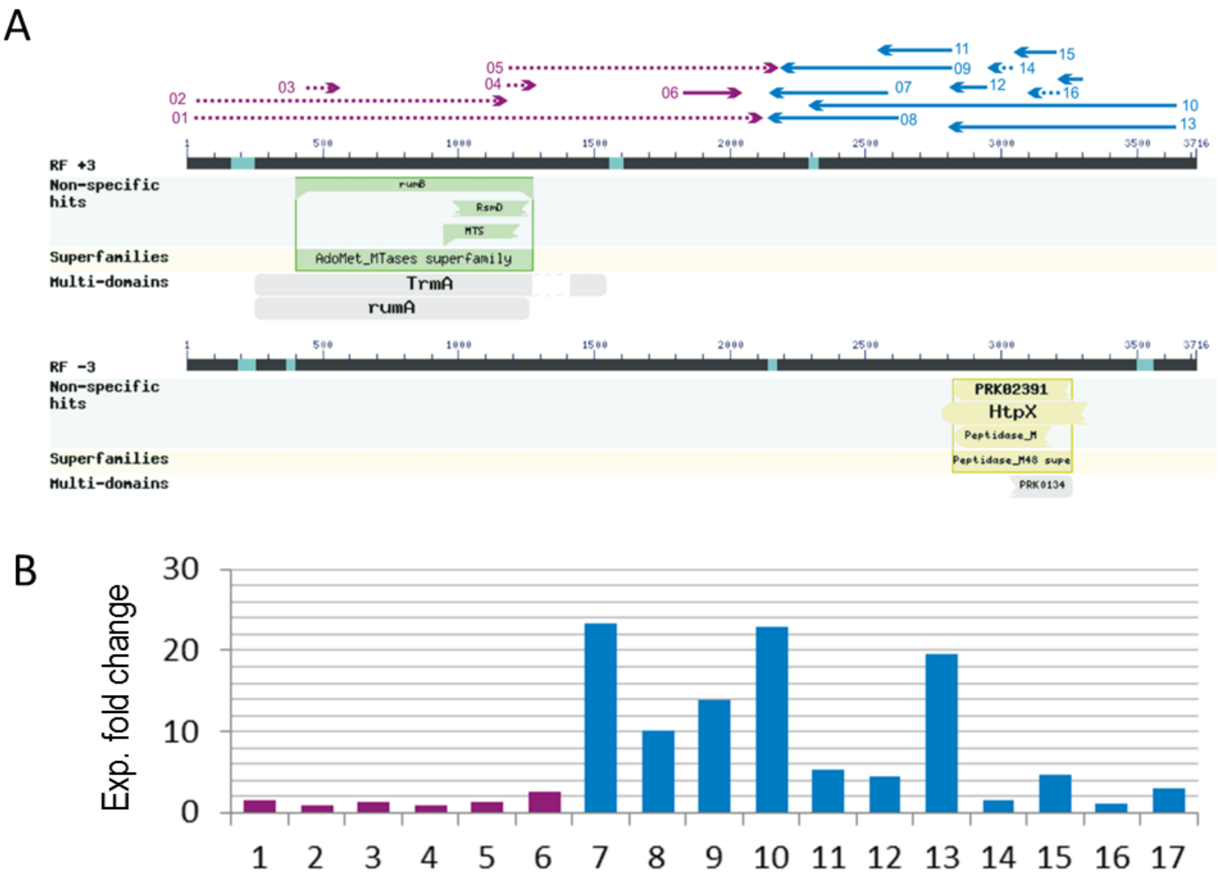

Supplementary Figure S3. Gene expression data for v.3 unigenes mapping v.5 primary transcript Nbv5tr6230285. A, Nbv5tr6230285 encodes two conserved domains, in different open reading frames: AdoMet-dependent methyltransferases superfamily (frame +3) and protease HtpX (frame -3). 17 v.3 unigenes with already defined sense strand match Nbv5tr6241943 sequence in two different orientations (presented in blue and purple color); B, Each of 17 v.3 unigenes is represented by a specific probe on Nb-105k microarray. Microarray data indicate different expression for 5' and 3' ends of Nbv5tr6241943, suggesting incorrectness of the transcript assembly. Solid lines mark unigenes for which 2-23-fold up-regulation was observed in leaves, compared to roots. Dotted lines indicate unigenes with <2-fold expression change.
